# Authors' Reply

**DOI:** 10.1371/journal.pcbi.0020026

**Published:** 2006-03-31

**Authors:** Eliot C Bush, Bruce T Lahn

## Authors' Reply

In their letter responding to our recent paper in *PLoS Computational Biology* [1,2], Keightley et al. provide a clear summary of the similarities and differences between the method used in their study [3] and that which was used in ours. They correctly point out that our study supports their conclusion that compared with rodents there has been an increase in sequence divergence rate in hominid noncoding sequences upstream of genes. They are also correct to say that the two studies are looking at slightly different populations of upstream noncoding sites. In their study, they calculate divergence in large blocks that include many different kinds of sites. Among these are nonfunctional sites, sites conserved among primates, and sites conserved among all mammals. As a result, their method can be thought of as broad but low resolution. In contrast, our method considers only sites that are likely to be conserved among all mammals, making it more restricted but higher resolution.

Our difference in focus allows us to make an important clarification of their earlier results. We find that despite the overall increase in divergence rate in hominid noncoding regions, significant constraint remains at some sites. In their letter, Keightley et al. acknowledge this point, but argue that such sites are likely to be relatively rare. To respond to this, we can calculate frequency values for different conservation scores from Table S1 of our paper. Windows with a score of 13 or higher constitute 2.7% of the total. (Sites next to these have an average hominid divergence of 0.0086, which is significantly constrained compared with the genome-wide average divergence rate of approximately 0.012.) This means that an average 10-kb upstream noncoding region would have hundreds of bases of this type. This is not a trivial number, and suggests that there are many highly conserved noncoding sites in hominids.

On the other hand, we agree that the method of Keightley et al. includes many sites that we ignore, and may reveal things that our method misses. These sites include functional sites that are not conserved in mouse or dog. Such sites might show an especially high divergence rate in hominids. It would be very interesting to quantitate the hominid divergence rate specifically at such sites, and compare it with the corresponding divergence rate in other mammals.

We would also like to take this opportunity to bring up a cautionary note that applies equally to both studies. Comparing human–chimpanzee divergence with mouse–rat divergence raises a number of complex technical issues because human–chimpanzee divergence is more than one order of magnitude smaller. Such issues include back mutations and varying contributions of polymorphisms and sequencing errors. To extend the work by Keightley et al. and our group, a “cleaner” future study might be to compare hominids with two closely related rodents (or other mammals) whose divergence is on par with human–chimpanzee divergence. It would be ideal to look at several such species pairs with varying population sizes, which may help one to assess whether the difference in divergence rate between hominids and rodents can be attributed to smaller historical population size in hominids.

Finally, whereas relaxation of selective constraint is a favored explanation for the higher divergence rate in hominids, it is by no means the only explanation. In the longer term, we also look forward to studies that quantitatively address the extent to which the higher hominid divergence rate is due to relaxation of functional constraint, positive selection, or other—as of yet poorly characterized— selective forces such as compensatory mutations.
